# Demographic and clinical characteristics of human bocavirus-1 infection in patients with acute respiratory tract infections during the COVID-19 pandemic in the Central Province of Sri Lanka

**DOI:** 10.1186/s12879-023-08312-x

**Published:** 2023-06-22

**Authors:** Shiyamalee Arunasalam, Thulani Pattiyakumbura, Sibra RM Shihab, Rohitha Muthugala, Faseeha Noordeen

**Affiliations:** 1 Diagnostic and Research Virology Laboratory, Department of Microbiology, Faculty of Medicine, University of Peradeniya, Peradeniya, 20400 Sri Lanka; 2 Virology Laboratory, National Hospital Kandy, Kandy, 20000 Sri Lanka

**Keywords:** Human bocavirus-1, Acute respiratory tract infections, Mono-/co-infections, Sri Lanka

## Abstract

**Background:**

Human bocavirus-1 (hBoV-1) was first detected in respiratory specimens in 2005. Due to high co-infection rates and prolonged shedding of the virus, the pathogenic role of hBoV-1 as a primary causative agent of respiratory infections is still under discussion. This study aimed to determine the prevalence of hBoV-1 infection in patients with acute respiratory tract infections (ARTIs) during the COVID-19 pandemic in the Central Province of Sri Lanka.

**Methods:**

A total of 1021 patients (Age 12 days to ≤ 85 years) with ARTI symptoms including fever, cough, cold, sore throat and shortness of breath within first 7 days of the illness were included. The study was carried out at the National Hospital, Kandy, Sri Lanka from January 2021 to October 2022. Respiratory specimens were tested to detect 23 pathogens including hBoV-1 using a real time PCR. Prevalence of hBoV-1 co-infections with other respiratory pathogens and distribution of hBoV-1 infection among different age groups were determined. Moreover, clinical and demographic characteristics of hBoV-1 mono-infection associated ARTI were compared with that of the hBoV-1 co-infections.

**Results:**

Respiratory infections were detected in 51.5% (526/1021) of the patients and of these 82.5% were mono- and 17.1% were co-infections. hBoV-1 was detected in 66 patients and this was the most prevalent respiratory virus associated with 40% co-infections. Of the 66 hBoV-1 positive patients, 36 had co-infections and of these 33 had dual and 3 had triple infections. Most of the hBoV-1 co-infections were identified in children aged 2-<5 years. hBoV-1 co-infections were most frequently detected with respiratory syncytial virus (RSV) and Rhino/ Entero viruses (Rh/EnV). No differences were observed in age, gender and clinical presentations in those with hBoV-1 mono- compared to co-infections. Intensive care admissions were less among hBoV-1 mono-infected than hBoV-1 co-infected patients.

**Conclusion:**

This study shows a prevalence of 12.5% for hBoV-1 infections in patients with ARTI. RSV and Rh/EnV were the most common co-infecting pathogens with hBoV-1. Clinical features of hBoV-1 mono-infections were not different to that of the hBoV-1 co-infections. Interactions between hBoV-1 and other respiratory pathogens need investigation to identify the role of hBoV-1 in clinical severity of co-infections.

## Background

Human bocavirus (hBoV) is a relatively new human virus closely related to bovine parvo- and canine minute viruses. The virus is classified in the genus *Bocaparvovirus* within the family *Parvoviridae* and was first described in 2005. hBoV is a single stranded non-enveloped DNA virus with four genotypes (hBoV 1–4) [1–3]. hBoV-1, strain of *Primate bocaparvovirus 1*, was first identified in respiratory specimens of Swedish children with acute respiratory tract infections (ARTIs) and the detection rate of hBoV-1 in patients with ARTI ranges from 1 to 56.8% [4–8]. Three other genotypes (hBoV-2, -3, -4) were subsequently detected in gastrointestinal samples and these are associated with gastroenteritis [9–11]. Although hBoV-1 is frequently detected in respiratory samples, its pathogenic role in respiratory infections is still being debated. hBoV-1 infections are characterized by rhinitis, cough, acute otitis media, and pharyngitis [12]. On the other hand, hBoV-1 is detectable in asymptomatic individuals and this is believed to be due to the persistence of hBoV-1 DNA in the nasopharynx for weeks to a year after the initial infection [13]. In addition to self-limiting upper respiratory tract infections, hBoV-1 also causes lower respiratory tract symptoms like bronchiolitis, exacerbations of asthma, pneumonia and respiratory distress [14]. Several cases of hBoV-1 associated severe respiratory illness requiring hospitalization and supplemental oxygen have been reported [15].

hBoV-1 is frequently detected along with other respiratory pathogens and studies report a high percentage of co-infections with frequencies ranging from 18 to 90% [16–19]. However, life threatening infections caused by hBoV-1 alone requiring ICU admission has also been reported. This is supported by correlating the viral load with disease severity in mono- infections and this finding suggests that the hBoV-1 can cause respiratory disease as a sole pathogen [5].

In Sri Lanka, data on epidemiology and clinical characteristics of hBoV-1 infections in ARTI are scanty. Thus the present study aimed to identify the prevalence of hBoV-1infection in patients with ARTI and to investigate the clinical characteristics of hBoV-1 mono- and co-infections.

## Methods

### Study design and setting

The study was conducted as a prospective descriptive study in a sample of patients with ARTI (Age 12 days to ≤ 85 years) from National Hospital, Kandy from January 2021 to October 2022. A total of 1021 patients with ARTI symptoms including fever (more than or equal to 38 ºC), cough, cold, sore throat and shortness of breath within first 7 days of the illness were selected for the study. Demographic and clinical data were extracted from the patients’ clinical notes.

The study was approved by the Ethical Review Committee of the Faculty of Medicine, University of Peradeniya, Sri Lanka (Permit No: 2021/EC/21) and informed consent was obtained from all subjects and/or their legal guardian(s) prior to sample collection. Moreover, informed consent was also obtained from the respective parent(s)/guardian(s) in the case of children. All methods including data and sample collection for the study were carried out in accordance with relevant guidelines and regulations.

### Collection and processing of samples

Respiratory specimens were subjected to nucleic acid extraction using the QIA Symphony nucleic acid extraction system (Qiagen, Germany) and the nucleic acid extracts were simultaneously tested for SARS-CoV-2 by real time RT-PCR (Altona, Real Star, Cat No: 821,015, Germany or Bioneer, Catalog No: nSCV-2112, South Korea) and other respiratory pathogens (influenza-A (inf-A), influenza-B (inf-B), influenza virus H1N1 pdm 09 (inf H1N1 pdm 09), respiratory syncytial virus-A (RSV-A), respiratory syncytial virus-B (RSV-B), human parainfluenza virus-1 (hPIV-1), human parainfluenza virus-2 (hPIV-2), human parainfluenza virus-3 (hPIV-3), human parainfluenza virus-4 (hPIV-4), human corona virus OC43 (hCoV OC43), humancoronavirus 229E (hCoV 229E), human coronavirus NL63/HKU1 (hCoV NL63/HKU1), Rhinovirus/Enterovirus (Rh/EnV), human adenovirus (hAdV), human metapneumo virus (hMPV), human bocavirus type-1 (hBoV-1) and four atypical bacteria *such as Mycoplasma pneumonia* (*M. pneumoniae*), *Chlamydophila pneumoniae* (*C. pneumoniae*), *Legionella pneumophilla* (*L. pneumophilla*), *Bordetella* species (*Bordetella spp*)) by a commercial real time PCR assay (Respifinder2SMART, Catalog No: PF2600-2 S, Netherlands) according to manufacturer’s instructions.

### Statistical analyses

Differences between clinical characteristics of hBoV-1 mono- and co-infections were analyzed using the statistical software Minitab, version 16.1. Categorical data were analyzed using Fisher’s Exact Test and the *p* value of < 0.05 was considered as significant.

## Results

We tested 1021 nasopharyngeal swabs / endotracheal secretions from patients with ARTI symptoms for 19 respiratory viruses including SARS CoV-2 and 4 atypical bacteria from January 2021 to October 2022. Of these patients, 60.2% were males with the mean age of 25 years and children to adult ratio was 1:1.05. Respiratory pathogens were detected in 526/1021 (51.5%) patients and of these 436/526 (82.5%) were positive for any of the respiratory pathogen and 90/526 (17%) were positive for more than one respiratory pathogens. Of those positive respiratory pathogen/s, 26.23% (138/526) had Rh/EnV, 18.44% (97/526) had RSV-A/B, 10.45% (55/526) had SARS CoV-2, 12.92% (68/526) had other hCoV 229E/ NL63/ HKU1/ OC43, 11.78% (62/526) had hPIV- 1–4, 12.92% (68/526) had inf-A/B, 4.75% (25/526) had hAdV, 3.8% (20/526) had hMPV and 3.8% (20/526) had bacteria *Bordetella spp*/ *L. pneumoniae*/ *M. pneumoniae*/ *C.pneumoniae*. hBoV-1 was detected in 66/526 (12.5%) patients. hBoV-1 was the most prevalent respiratory virus associated with co-infections (40%, 36/90). Male to female ratio was 1:1.2 for hBoV-1 infected patients whose age ranged from 45 days to 85 years. hBoV-1 infection was more prevalent in children (84.8%) than that in adults. Of the 66 hBoV-1 positive patients, 36 (54.5%) were co-infected with other respiratory pathogens including viruses (92%) and atypical bacteria (8%) including RSV, hAdV, hMPV, inf-A virus, hCoV 229E, SARS-CoV-2, Rh/EnV, *M. pneumoniae* and *Bordetella spp*.

**Fig. 1:**
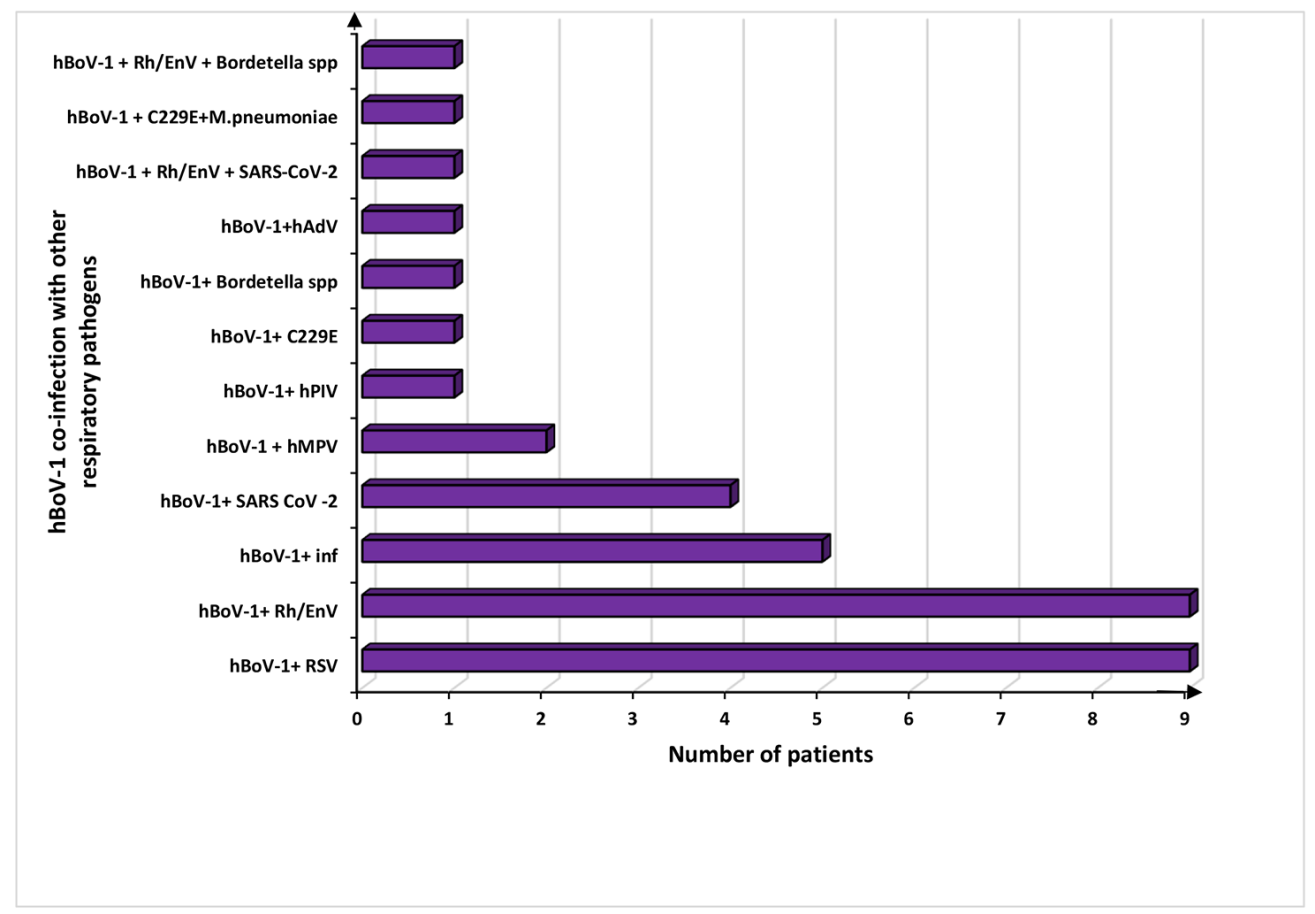
Prevalence of hBoV-1 co-infections with other respiratory pathogens (n = 36). hBoV-1 co-infections were commonly noted with RSV and Rh/EnV compared to other respiratory pathogens. hBoV-1 - human bocavirus − 1, Rh/EnV- rhino/ entero viruses, C229E - human corona virus 229E, hAdV - human adeno virus, hPIV - human parainfluenza virus, hMPV - human metapneumo virus, RSV - respiratory syncytial virus and *M. pneumonia* - *Mycoplasma pneumoniae*

**Fig. 2:**
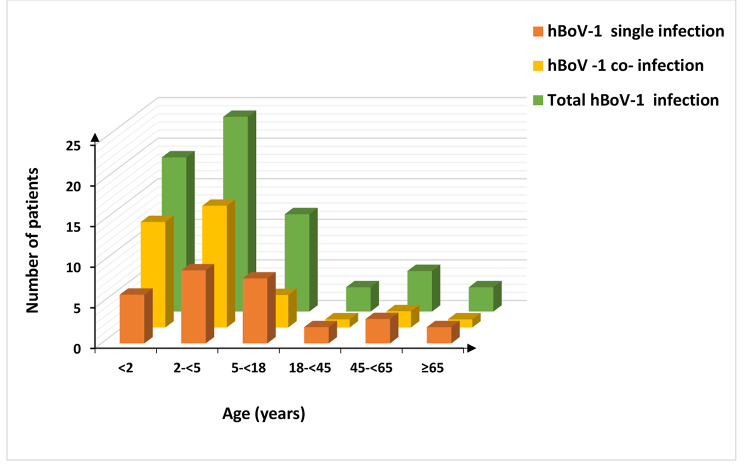
Distribution of hBoV-1 infection in different age groups. The Fig shows the distribution of hBoV-1 mono-, co- and total infections in different age groups. hBoV-1 co-infections were identified more commonly in children aged between 2-<5 years. hBoV-1 - human bocavirus-1

Of the 36 hBoV-1 co-infected patients, 33 had dual infections while 3 others had triple infections. Of those with dual infections, 32 had co-infection with other respiratory viruses and one with *Bordetella spp*. Of the three children with triple infections, one had co-infection with two different respiratory viruses while the other two patients had infection with a virus and a bacteria (Fig. 1). Moreover, hBoV-1 more commonly co-infected with RSV and Rh/EnV compared to other respiratory pathogens. Most of the hBoV-1 co-infections were detected in children aged 2-<5 years (24/36; 66.6%) (Fig. 2).

Clinical and demographic characteristics of hBoV-1 mono-infection associated ARTI were compared with that of the hBoV-1 co-infections and no significant differences were noted between clinical and demographic characteristics of hBoV-1 mono- and co-infections (Table 1). The most common symptoms of hBoV-1 infected patients were fever (45/66, 88.8%) and cough (30/66, 45.4%). Of the 526 patients positive for respiratory pathogens, 50 (9.5%) required intensive care and of these, 20 (40%) patients had hBoV-1 infection including 11 (22%) patients with co-infections. All hBoV-1 co-infections were identified in children except one in a post-partum woman.

**Table 1:** Demographic and clinical characteristics in hBoV-1 mono-and co-infections

	hBoV-1 mono- infections (n = 30)	hBoV-1 co-infections (n = 36)	*P*
Demographic characteristics			
Males	10 (33.3%)	21 (58.3%)	0.051
Age ± SD (Years)	20 ± 23	9 ± 19	0.492
**Clinical characteristics**			
Fever	19 (63.3%)	26(72.2%)	0.596
Cough	15 (50%)	15 (41.6%)	0.212
Sore throat	4 (13.3%)	4 (11.1%)	0.492
Shortness of breath	10 (33.3%)	10 (27.7%)	0.788
Diarrhoea	3 (10%)	3 (8.3%)	1
Co-morbidities	6 (20%)	7 (19.4%)	1
Admission to intensive care unit	9 (30%)	11 (30.5%)	1

Numerical data are expressed as mean and categorical variables are expressed as percentages and proportion. Variables were compared by Fisher’s exact test. No statistically significant difference was noted between the two groups (*p* < 0.05). *hBoV-1* human - bocavirus-1.

## Discussion

The present study is the first large-scale study with comprehensive clinical details on hBoV-1 infection in patients with ARTI symptoms in the Central Province of Sri Lanka. Here, we used commercially available real time PCR assays to test for 23 respiratory pathogens including viruses and atypical bacteria in 1021 patients.

Despite the COVID-19 control measures, the detection rate of respiratory pathogens was 51.5% in the current study and this rate of detection is higher than the other studies reported from Sri Lanka by Divarathna et al. (D3 Ultra Respiratory Virus Screening and ID Kit, Diagnostic Hybrids, USA – Catalog No:01-010000.v2 which detects RSV A, B, inf-A, B, hAdV, hPIV1-3) [20], Jayaweera et al. (D3 Ultra 8 DFA Respiratory Virus Screening & Identification Kit, Diagnostic Hybrids, USA – Catalog No: I-01-110000 which detects RSV, Inf A, B, hAdV, hPIV1-3 and hMPV [21] and Muthulingam et al., Imagen™, UK – Catalog number k6121 which detects RSV A, B, hAdV, infA, B, hPIV 1–3) [22] and in these studies the prevalence was 47.4%, 39.4% and 32.3%, respectively. These studies used antigen detection by immunofluorescence assay (IFA) in children and these studies were conducted prior to the pandemic. However, the PCR is more sensitive compared to the IFA and that the current study has detected a wide spectrum of 23 respiratory pathogens. The multiplex real time PCR assay has the advantage of detecting a wide spectrum of respiratory pathogens with higher sensitivity compared to the IFA.

The present study detected a co-infection rate of 17%, which is lower than that reported by other studies done in patients with a wide age range in those the co-infection rate is 32.76% and 37%, respectively [3, 23]. Almost all pathogens had co-infections with another respiratory pathogen, however, the hBoV-1 was associated with high rates of co-infection in our study and this finding is similar to a study reported by Finianos et al. in children [24].

As previously reported, the worldwide prevalence of hBoV-1 in respiratory infections ranged from 1 to 56.8% and our prevalence rate is in line with the previous findings [4, 8, 25]. The prevalence of hBoV-1 was 12.5% in our study and this is significantly higher than that reported by Madi and Al-Adwani (1.9%) who tested patients with ARTI in Kuwait and China (4.6%) [19]. In contrast to the finding of Ghietto et al., our prevalence rate (12.5%) is slightly lower than that reported in Argentina (22.7%) in infants and adults with lower ARTI [14]. Regional and temporal variations, differences in the study sample and the differences in sample collection might also have contributed for variations in the prevalence [19]. A higher prevalence of 36.3% hBoV-1 infection was noted in children between 2 to ≤ 5 years in the current study compared to children < 2 years. These results show that children within 2 to ≤ 5 years are more susceptible to hBoV-1 infection and this might be due to the waning of maternally acquired antibodies against the virus. Lower prevalence of hBoV-1infection in adults, as shown in other studies [8, 17, 26, 27] also, may be due to adulthood immunity against hBoV-1 infection.

hBoV-1 mono-infection was detected in 45.5% of the infected patients in the current study sample and this mono-infection rate is higher than that reported by Arwa A. Bagasi (23.2%) in patients in the United Kingdom [2]. Although the average day of detection of hBoV-1 mono-infection from the onset of symptoms is four days in the current study sample, we still cannot rule out the possibility that all 45.5% of hBoV-1 mono- infections were caused by a recent hBoV-1 infection or hBoV-1 viral shedding from a previous infection as hBoV-1 can persist for weeks to months following an infection [28, 29].

hBoV-1 co-infection rate with other pathogens was 54.5% in the current study and this rate of co-infection is lower than that reported by Calvo et al. (75%) in children [30] and higher than the study by Lee et al. in adults (47.6%) [31]. hBoV-1 co-infections with viruses are comparatively higher than that with bacteria. In our study, atypical bacteria were detected in 8% of hBoV-1 positive patients, and the bacterial infection rate was lower than that observed in a previous study done by Cia et al. in adults [32]. Of the hBoV-1 co-infections, the highest percentage of hBoV-1 co-infection was noted with RSV and Rh/EnV in the current study as reported elsewhere too [14]. Although hBoV-1 co-infections were more frequently detected in children (88.8%) than adults (11.2%) in the current study, a difference was not detected statistically (*p* = 0.492).

The most predominant symptom among hBoV-1 mono-and co-infections was fever (63.3% and 72.2%, respectively) followed by cough (50% and 41.6%, respectively). In the current study, patients with hBoV-1 mono-infection had similar symptoms to those with co-infections and this is in agreement with the findings of Madi and Al- Adwani [19]. However, 9 mono- and eleven co-infected patients required intensive care and this finding was similar to a study done in the United Kingdom, where the intensive care admissions were considerably higher among HBoV-1 co-infected patients than hBoV-1 mono-infected patients [2]. In our study, all hBoV-1 co-infected patients were children except a post-partum woman and this reflects the burden of hBoV-1 co-infection in high risk groups, yet further studies are needed to understand the severity of hBoV-1 as a co-infecting respiratory pathogen. On the other hand, as previously also pointed out hBoV-1 DNA can be detected for weeks to several months following an acute infection and this may complicate the interpretation of co-infections following the test results.

## Conclusion

In summary, hBoV-1 was prevalent in 12.5% of the ARTI patients in whom hBoV-1 was detected alone or with other respiratory pathogen/s. hBoV-1 was the most common virus co-infecting with other respiratory pathogens. RSV and Rh/En were the most common co-infecting respiratory pathogens with hBoV-1. Most of the hBoV-1 co-infections were identified in children aged 2-<5 years. No differences were observed with clinical characteristics of patients with hBoV-1 alone or in combination with other respiratory pathogens. Intensive care admissions were considerably higher among hBoV-1 co-infected patients than those with hBoV-1 mono-infection. However, the interaction between hBoV-1 with other respiratory pathogens needs further investigation to identify the role of hBoV-1 in clinical severity of co-infections.

## Data Availability

The data and materials supporting the conclusions of the study are available from the corresponding author on reasonable request.

## Abbreviations

ARTI: Acute respiratory tract infection
hAdV: Human adeno virus
hBoV-1: Human bocavirus-1
hMPV: Human metapneumovirus
hPIV: Human parainfluenza virus
IFA: Immunofluorescence assay
Rh/EnV: Rhino/ Entero viruses
RSV: Respiratory syncytial virus.
